# Stroke after Cardiac Surgery: A Risk Factor Analysis of 580,117 Patients from UK National Adult Cardiac Surgical Audit Cohort

**DOI:** 10.3390/jpm14020169

**Published:** 2024-01-31

**Authors:** Laura Asta, Daniele Falco, Umberto Benedetto, Annamaria Porreca, Fatma Majri, Gianni D. Angelini, Stefano Sensi, Gabriele Di Giammarco

**Affiliations:** 1 Department of Cardiac Surgery, Tor Vergata University Hospital, 00133 Rome, Italy; astalaura92@gmail.com; 2 Division of Cardiac Surgery, SS Annunziata Hospital, 66100 Chieti, Italy; daniele.falco@studenti.unich.it; 3 Department of Neuroscience, Imaging and Clinical Sciences, Cardiac Surgery Department, University “G. d’Annunzio” Chieti-Pescara, 66100 Chieti, Italy; umberto.benedetto@asl2abruzzo.it (U.B.); stefano.sensi@unich.it (S.S.); gabriele.digiammarco@unich.it (G.D.G.); 4 Department of Medical, Oral and Biotechnological Sciences, University “G. d’Annunzio” Chieti-Pescara, 66100 Chieti, Italy; 5 Department of Protection and Prevention, SS Annunziata Hospital, 66100 Chieti, Italy; fatma.majri@asl2abruzzo.it; 6 Department of Cardiac Surgery, University of Bristol, Bristol BS8 1QU, UK; g.d.angelini@bristol.ac.uk

**Keywords:** stroke, cerebral vascular accident, cardiac surgery

## Abstract

Cerebrovascular accident is the most ominous complication observed after cardiac surgery, carrying an increased risk of morbidity and mortality. Analysis of the problem shows its multidimensional nature. In this study, we aimed to identify major determinants among classic variables, either demographic, clinical or type of surgical procedure, based on the analysis of a large dataset of 580,117 patients from the UK National Adult Cardiac Surgical Audit (NACSA). For this purpose, univariate and multivariate logistic regression models were utilized to determine associations between predictors and dependent variable (Stroke after cardiac surgery). Odds ratios (ORs) and 95% confidence intervals (CIs) were constructed for each independent variable. Statistical analysis allows us to confirm with greater certainty the predictive value of some variables such as age, gender, diabetes mellitus (diabetes treated with insulin OR = 1.37, 95%CI = 1.23–1.53), and systemic arterial hypertension (OR = 1.11, 95%CI = 1.05–1.16);, to emphasize the role of preoperative atrial fibrillation (OR = 1.10, 95%CI = 1.03–1.16) extracardiac arteriopathy (OR = 1.70, 95%CI = 1.58–1.82), and previous cerebral vascular accident (OR 1.71, 95%CI = 1.6–1.9), and to reappraise others like smoking status (crude OR = 1.00, 95%CI = 0.93–1.07 for current smokers) or BMI (OR = 0.98, 95%CI = 0.97–0.98). This could allow for better preoperative risk stratification. In addition, identifying those surgical procedures (for example thoracic aortic surgery associated with a crude OR of 3.72 and 95%CI = 3.53–3.93) burdened by a high risk of neurological complications may help broaden the field of preventive and protective techniques.

## 1. Introduction

Stroke after cardiac surgery (SACS) means a clinically evident or radiologically demonstrable neurological event, ischemic or hemorrhagic, occurring within the first month after cardiac surgery, according to the definitions of the Society for Neuroscience in Anesthesiology and Critical Care (SNACC) [1]. The clinical manifestations are extremely variable: they can range from sensorial, cognitive, and behavioral alterations, visual alterations or aphasia, to focal neurological deficits.

This major postoperative complication continues to have a high negative impact on mortality and morbidity. In particular, it increases the costs of both inpatient and follow-up care [2], which are the most frequent causes of rehospitalization [3]. In this regard, Alrifai et al. highlighted that certain characteristics predispose patients suffering from SACS to a greater risk of rehospitalization, including female gender, age above 65, nonwhite race, lower household income, and increased comorbidities [4].

Its occurrence is closely related to the type of surgical procedure; in patients undergoing coronary artery bypass graft surgery (CABG), the estimated incidence varies from 1 to 6% and increases up to 10% in aortic valve surgery [5]. Furthermore, it has been established that the mortality of SACS is higher than the same neurological event in patients who have not undergone surgery [6]. The progressive increase in the age of patients undergoing cardiac surgery has certainly led to an increase in the frequency of this complication [7], although it is an event with a multifactorial and not always defined etiology.

Therefore, our study aims to investigate the major risk factors by retrospectively analysing the UK National Adult Cardiac Surgical Audit (NACSA) data to stratify the risk as much as possible when planning surgery. We analyzed demographic variables such as age, gender, body mass index (BMI), smoking status, clinical-like systemic arterial hypertension, diabetes mellitus, extracardiac arteriopathy, previous cerebral vascular accident, left ventricular ejection fraction, preoperative atrial fibrillation; finally, the risk was correlated with the type of cardiac surgery (valve, surgery for thoracic aortic disease, and coronary artery bypass grafts operation, CABG).

We believe that statistical analysis of these variables can, on the one hand, better stratify the risk of patients undergoing cardiac surgery and, above all, based on the type of surgery, encourage the use of techniques and strategies that can reduce the risk.

## 2. Materials and Methods

### 2.1. Study Population

This retrospective study uses data from the United Kingdom (UK) National Adult Cardiac Surgical Audit (NACSA) of 580,117 patients undergoing cardiac surgery from January 1995 to December 2015. This dataset collects pre-, peri-, and postoperative demographic and clinical information for all major cardiac surgery procedures on adults performed in the UK [8]. The key function of NACSA is to benchmark surgical practice. The database managers validate data entered locally by surgeons; therefore, this register undergoes regular robust validation and control procedures to achieve and maintain high-quality standards of information. The pre- and intraoperative variables considered, according to the recent literature, to have the greatest impact were analysed in the study population.

### 2.2. Statistical Analysis

The data are analyzed using descriptive statistics appropriate for the nature of the variables. Continuous variables are presented with median [q1 = first; q3 = third] quartile Normality distribution is tested using Shapiro–Wilk. Categorical data are presented as absolute frequencies and percentages. The bar graph was used to depict the incidence of SACS at 5 years. The univariate and multivariate logistic regression models determine associations between the endpoint (SACS), patients’ characteristics and procedure factors. Crude and adjusted odds ratios (ORs) with their 95% confidence interval (CI) were calculated. The multivariate logistic regression model was internally validated using 80% of the sample as the training set and 20% as the test set. Thus, an ROC curve was built, and the Area under the curve (AUC) with its 95%CI was reported. All statistical analyses were performed using the R statistical environment (version 4.1; R Foundation for Statistical Computing, Vienna, Austria). All *p*-values were two-tailed, and a *p*-value ≤ 0.05 indicated a statistically significant association.

## 3. Results

**Figure 1:**
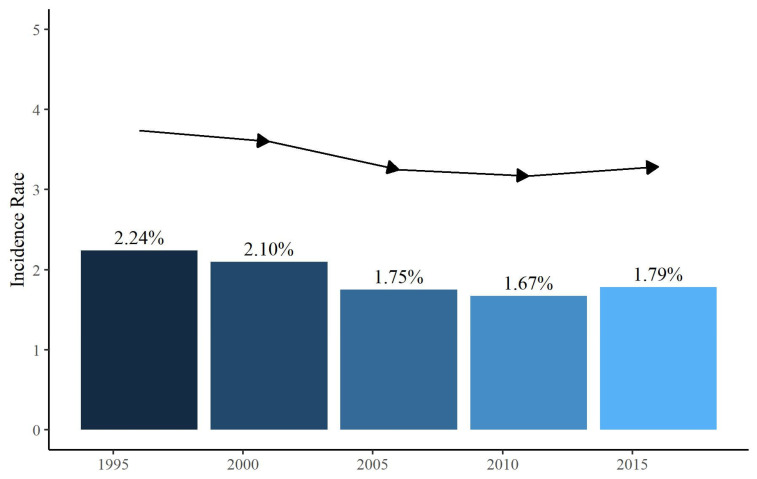
Incidence rate of Stroke After Cardiac Surgery (SACS) at 5 years (from 1995 to 2015).

Out of 580,117 patients who underwent cardiac surgery reported in the database, the total number of patients who presented with SACS (clinically evident or radiologically demonstrable neurological event, ischemic or hemorrhagic, occurring within the first month after cardiac surgery) was 10,757 (1.85%) (Figure 1).

The patient characteristics analyzed were age, gender, and BMI. The preoperative risk factors examined were systemic arterial hypertension, diabetes mellitus, smoking status, extracardiac arteriopathy, previous cerebral vascular accident, left ventricular ejection fraction, and pre-operative atrial fibrillation. Finally, the risk of SACS was related to the type of surgical procedure (valve, thoracic aortic surgery, and CABG operation). For each variable, incidence was studied in patients with SACS and those who did not present with neurological complications. Furthermore, ORs and 95%CI were calculated from univariate logistic regression models to assess important risk factors for SACS (Table 1).

### 3.1. Patient Characteristics

The median age of patients without SACS was 67.7 years, while that of patients with SACS was 71.8 years. However, the proportion of women with SACS (32.6%, *n* = 3504) was higher than the female population without SACS (27.1%, *n* = 154,169), with a male gender OR of 0.77 and 95% CI of 0.74–0.80, and a female gender OR of 1.30 [1.25;1.36]. Patients with SACS had an average BMI of 26.8, unlike patients without neurological complications who had a median BMI of 27.6.

### 3.2. Risk Factors

Systemic arterial hypertension (treated or blood pressure (BP) > 140/90 mmHg on >1 occasion before admission) was present in 68.6% (*n* = 7269) of SACS patients, with an OR of 1.19 and 95% CI of 1.14–1.24. Diabetes mellitus was divided by treatment: diet alone, oral therapy, and insulin therapy. These were present in the group of patients with SACS in 4.07% (*n* = 438), 11.6% (*n* = 1249), and 6.02% (*n* = 648) of cases, respectively; among the three groups, the highest OR value was attributed to the category treated with insulin therapy (OR of 1.12 and 95% CI of 1.03–1.21). Therefore, both systemic arterial hypertension and diabetes mellitus (especially if treated with insulin therapy) appear to be risk factors for SACS.

On the other hand, smoking status indicated no statistically significant difference in the two groups. Smoking status was divided into two categories: ex-smokers and current smokers. Patients with a history of smoking (ex-smokers) were 51.8% (*n* = 5465) of patients affected by SACS, while patients with a current smoking habit constituted 10.6% (*n* = 1122) with ORs of 0.97 and 1.0, and 95% CIs of 0.97 [0.94;1.02] and 1.00 [0.93;1.07], respectively.

Extracardiac arteriopathy refers to the presence of an atherosclerotic plaque documented via color Doppler ultrasound responsible for a significant hemodynamic effect. Previous cerebral vascular accidents included all clinical manifestations of cerebrovascular accident: ischemic, hemorrhagic stroke, and transient ischemic attack.

Extracardiac arteriopathy and previous cerebral vascular accidents were significant risk factors for SACS. In fact, for these two variables, present in 19.4% (*n* = 2091) and 7.32% (*n* = 787) of patients affected by SACS, respectively, and ORs of 1.94 and 2.32, and 95% CIs of 1.94 [1.85;2.04] and 2.32 [2.16;2.50], were calculated.

Left ventricular ejection fraction (LVEF) was divided into three categories: good (LVEF ≥ 50%), fair (LVEF 30–50%), and poor (LVEF < 30%). Patients with fair and poor LVEF were 28.2% (*n* = 2976) and 7.52% (*n* = 793) of patients with SACS, respectively. The univariate analysis revealed ORs of 1.31 and 1.48 and 95% CIs of 1.25–1.37 and 1.37–1.60. Therefore, the progressive reduction in LVEF was associated with an increased risk of SACS. Similarly, the presence of preoperative atrial fibrillation increased the risk of SACS. By preoperative atrial fibrillation, we meant a positive history of first-onset, paroxysmal, persistent, long-lasting persistent, and permanent atrial fibrillation. In fact, in patients suffering from this postoperative complication, 15.5% (*n* = 1516) had a positive history of atrial fibrillation. The univariate analysis calculated an OR of 1.61 with a 95% CI of 1.52–1.70.

### 3.3. Type of Procedure

SACS was related to the type of procedure the patient underwent.

Cardiopulmonary bypass has emerged as an independent risk factor for the onset of SACS. Cardiopulmonary bypass was used in almost all the operations of patients with SACS, i.e., 95.6% (*n* = 10,080), and for this variable, an OR of 2.38 was calculated with a 95% CI of 2.17–2.62.

Among the various surgical procedures, thoracic aortic surgery was considered the greatest risk for SACS. A total of 15.8% (*n* = 1696) of patients affected by SACS underwent thoracic aortic surgery. The OR calculated for this variable was 3.72, with a 95% CI of 3.53–3.93. Aortic valve surgery followed, with an incidence of 36.4% (*n* = 3918) in the group affected by SACS and a 95% CI of 1.48–1.60. At the same time, mitral valve and tricuspid valve surgery were performed, respectively, in 16.3% (*n* = 1749) and 3.09% (*n* = 332) of SACS patients with respective ORs of 1.49 and 1.39, and 95% CIs of 1.42–1.57 and 1.24–1.55.

On the contrary, CABG operation, performed in 58.7% (*n* = 6312) of patients with SACS, showed an OR of 0.59 and 95% CI of 0.57–0.62 in the univariate analysis. Therefore, based on our analysis, it does not constitute a significant risk factor for SACS.

Finally, the multivariate logistic regression model on the training set adjusted all odds ratios (ORs) and 95%CIs (Table 2). In the multivariate logistic regression, all variables under study preserve their verse as protective or risk factors except for tricuspid valve surgery. Although this variable is a risk factor in the univariate analysis, in the multivariate, the OR changes direction (OR = 0.88, 95%CI: 0.75–1.04, *p* = 0.133). This relationship may be because there are unconsidered confounding variables, although the relationship is not statistically significant.

**Figure 2:**
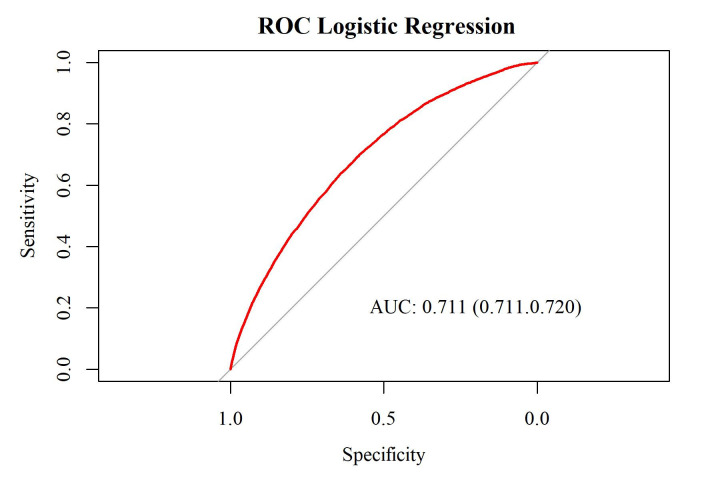
The ROC-curve and AUC (95% confidence interval) for the multivariate logistic regression model on the test set.

Figure 2 shows the multivariate logistic regression model’s performance on the test set, which comprises 20% of the cohort. An AUC greater than 0.9 indicated excellent diagnostic efficacy. An AUC between 0.7 and 0.9 indicated good diagnostic efficacy. An AUC between 0.5 and 0.7 indicated poor diagnostic efficacy. An AUC of no more than 0.5 indicated the lack of a diagnostic value of the marker using the method suggested by DeLong et al. [9]. In this study, the predictive performance of AUC = 0.711 (95%CI = 0.711–0.720) indicated a good prediction of SACS.

## 4. Discussion

SACS remains one of the complications that most compromises the outcome of patients undergoing cardiac surgery, and although it is rare, the consequences remain severe. The incidence emerging from our study (1.85%) is lower than those reported by others [10,11] but similar to the trend of the annual report of the Society for Cardiothoracic Surgery in Great Britain and Ireland, which documents a further decrease in incidence from 1.5% to 1% in the period between 2004 and 2008 [12]. On the contrary, Jonsson et al. in SWEDEHEART (Swedish Web System for Enhancement and Development of Evidence-Based Care in Heart Disease Evaluated According to Recommended Therapies) highlighted that the average annual SACS incidence in patients undergoing CABG operations was 1.2%, with a variation between 0.6% and 1.7% in the period under study (2006–2017), and that this, in the unadjusted logistic regression models and adjusted only for age and sex, did not amount to any statistically significant change, while in the multi-adjusted model, there was a statistically significant but minimal reduction. Therefore, there is no clear direction for SACS’s increasing or decreasing trend. This confirms that the techniques currently used are insufficient and that the problem is still of great scientific interest [13]. However, in evaluating the incidence of SACS, it is necessary to underline that there is a submerged part associated with a silent clinical manifestation that emerges in radiological evaluations carried out subsequently.

Certain factors, such as cerebral hypoperfusion, hyperthermia, systemic inflammation or hypoxemia due to prolonged anemia, related to the use of cardiopulmonary bypass (CPB), are themselves determinants of stroke [14], as confirmed by our study in which the use of cardiopulmonary bypass was associated with an increased risk of SACS.

It should be noted, however, that the type of surgery significantly affects the incidence of this complication.

While tricuspid valve and mitral valve surgery are associated with ORs of 1.39 and 1.49, respectively, aortic valve surgery and even more so surgery on ascending thoracic aorta, with ORs of 1.54 and 3.72, respectively, are confirmed to be the surgical procedures most associated with risk of SACS. In addition, Messé and colleagues demonstrated using MRI evaluation that in patients undergoing aortic valve surgery, silent cerebral infarction occurred in more than half of the cases without clinical evidence of stroke [15].

A calcified aortic wall and the related risk of embolization during surgical maneuvers (valve removal, aortic clamping) are major mechanisms responsible for SACS [16]. Different methods can be used to define the presence of an atheroma, from simple digital palpation of the aortic wall through epiaortic ultrasound to transesophageal echocardiography.

Zhao et al. demonstrated through a meta-analysis study that included the evaluation of 37,720 patients that the risk of SACS was associated with a 78% reduction in patients treated without aortic manipulation compared to CABG with aortic cross-clamping, 66% compared to CABG with off-pump with a partial clamp, and 52% compared to off-pump with the clampless Heartstring device [17].

In addition, using protective equipment can be a valid strategy to reduce the incidence of stroke [18]. Currently, the device is only used in patients with TAVI (Transcatheter Aortic Valve Implantation) indications, but it is hoped that it can also be used in cardiac surgery.

Regarding the surgical access route, Bozhinovska et al. have also demonstrated through transcranial Doppler evaluation that there are no significant differences between surgical approaches (ministernotomy or minithoracotomy) in the incidence of microembolisms [19].

Based on the above, it is understandable how off-pump CABG, along with the aorta no-touch technique, is associated with a lower rate of SACS [20,21].

The median age of patients with postoperative stroke was significantly higher than the non-stroke group, demonstrating that age is a significant non-modifiable risk factor [22]. Another similar factor is the female gender. It is now established that women have a higher general risk of stroke due to genetic, hormonal, and neurovascular characteristics [23]. Furthermore, since atherosclerotic processes speed up after menopause, the risk of SACS increases more with age in female patients.

Systemic arterial hypertension and diabetes mellitus, which are two independent stroke risk variables, share the same pathogenetic mechanism regarding the development of cerebral microcirculatory damage [24]. Diabetes mellitus also causes endothelial damage with significant alteration in tissue perfusion at the coronary level with the development of ventricular dysfunction [25].

From our analysis, it emerged that the progressive reduction i left ventricular ejection fraction is associated with an increased risk of stroke with a significant difference in risk for values below 30% (LVEF < 30%). This correlation is explained by cerebral hypoperfusion linked to low output but also by the increased risk of thromboembolism in patients with ventricular dysfunction [26]. Aljaber and colleagues, in an echocardiographic study of 82 patients with an average LVEF of 30%, showed the presence of thrombus within the left ventricle in 6.01% of patients [27].

Extra cardiac arteriopathy is another risk factor that emerged as significant. Extra cardiac arteriopathy is a marker of a widespread atherosclerotic process and shares the same risk factors with stroke (advanced age, arterial hypertension, diabetes mellitus); therefore, it seems reasonable that its presence, understood as a disease of the epiaortic vessels or peripheral arterial disease, considerably increases the risk of SACS. Several studies, in agreement with what we have demonstrated, have highlighted how unilateral, and even more so bilateral, stenosis of the internal carotid artery (ICA) can be considered an independent risk factor for SACS [28,29]. The etiopathogenetic hypotheses refer to two main mechanisms: the thromboembolic event originating from an unstable plaque and systemic hypoperfusion. In particular, Raffa et al. hypothesized that the carotid intima-media thickness can also be used to assess the risk of SACS. Therefore, the preoperative assessment via color Doppler ultrasound (or even better via chest CT scan without contrast dye) has a fundamental role not only in the assessment of the ICA but also in the evaluation of the presence of atherosclerotic plaques at the level of the ascending aorta and the aortic arch [29].

The presence of preoperative atrial fibrillation is associated with a significant risk of SACS negatively affecting patient outcomes [30]. The use of the CHA2DS2-VASc score, which defines the risk of stroke and appropriate anticoagulant therapy, includes a careful evaluation using trans-esophageal echocardiography, and highlights the presence of thrombus in the left atrial appendage, can reduce the operative risk. In addition, surgical closure of the appendage and the use of bipolar radiofrequency ablation around the pulmonary veins and in the posterior wall of the left atrium produced excellent results in treating preoperative atrial fibrillation [31] with a significant reduction in SACS [32].

Among those preoperative variables analyzed, previous cerebral vascular accident (ischemic, hemorrhagic stroke or transient ischemic attack) was found to have the highest risk of occurrence. A previous neurological episode is an indication of an already existing cerebral vasculopathy, which predisposes patients to the onset of new events. Others share this figure in the literature. Magedanz and colleagues, in their retrospective cohort study, showed that a positive history of stroke was associated with the risk of a new episode with an OR of 3.4 (95% CI 2.2–5.2, *p* < 0.001) [33]. Regarding the same risk of a repeated ischemic episode, D’Ancona and colleagues reported an OR of 2.15 in 9916 patients undergoing CABG, hypothesizing that careful monitoring of cerebral perfusion pressures and prevention of perioperative hypotension and low-volume situation may reduce the risk of new ischemic episodes [34].

In addition, in our analysis, an increase in BMI was not associated with a significant risk of SACS and the mean BMI value in patients who did not have SACS was higher than those who did (27.6 vs. 26.8). Slightly higher risk rates have emerged from other studies (Santos and colleagues report an OR of 1.19, a 95% CI of 0.71–2.0, and *p* = 0.59 [35]) but not to the extent that obesity can be considered as one of the major risk factors for the onset of SACS. Moreover, the obesity paradox documents how obese patients in whom a neurological complication occurs have a better outcome than the rest of the population due to mechanisms that are still not fully understood [36].

Similarly, and in line with other studies [37], smoking status is not one of the major risk factors for neurological pathology, remaining instead an incontrovertible risk factor for the onset of pulmonary complications [38].

Analysis of the variables above can have a decisive role not only in preoperative risk stratification but also in the study of the long-term survival of patients affected by SACS. Although numerous studies evaluate the incidence of SACS in relation to the main dependent and independent risk factors, the relationship between these and long-term survival is still lacking in certain respects. In this regard, Wagner et al. highlighted how certain risk factors negatively affect the 1-year survival of patients with SACS. Among these, renal failure, postoperative coma, extracardiac arteriopathy, age, prolonged ventilation, reoperation for bleeding, and insulin-treated diabetes emerged as the major predictors for survival in stroke patients compared with non-stroke patients in the past year [39].

Finally, after the risk stratification of SACS and, therefore, the identification of patients who are most likely to present this neurological complication, much remains to be done not only for post-stroke treatment techniques but, above all, for prevention ones.

Jin et al. proposed using neuroprotectors such as exenatide, a postsynaptic density protein 95 inhibitor that counteracts oxidative stress, in patients at high risk of SACS undergoing surgical procedures that predispose patients to an increased risk. Nerenetide has demonstrated its maximum efficacy when administered 1.5 h after stroke onset in primates, but since this timing is not feasible in clinical activity, they proposed preventive use [40].

The limitations of our study include a failure to subdivide the SACS event into ischemic or hemorrhagic. This subdivision, associated with the surgical procedure performed, could clarify some etiological aspects. Furthermore, the data available do not allow us to understand the impact of the neurological complication on patient survival subject to statistical analysis.

## 5. Conclusions

In conclusion, our analysis of such a large dataset allows us to confirm with greater certainty the responsibility of some already known factors such as advanced age, systemic arterial hypertension, and diabetes mellitus, to underline the importance of others like preoperative atrial fibrillation, extracardiac arteriopathy, previous cerebral vascular accidents, and the reduction in % left ventricular ejection fraction, and to correlate the risk to the type of surgical procedure in order to stratify preoperative patients as much as possible and to adopt all appropriate preventive and control measures against this ominous complication.

## Figures and Tables

**Table 1:** Crude odd ratios (ORs) and 95% confidence intervals (CIs) from univariate logistic regression models to assess important risk factors for SACS.

Variables	All Patients	No SACS	SACS	OR [95%CI]	*p*-Value
	*n* = 580,117	*n* = 569,360	*n* = 10,757		
Age, years	67.8 [59.7;74.5]	67.7 [59.6;74.4]	71.8 [64.4;77.1]	1.03 [1.03;1.03]	<0.001
BMI, kg/m^2^	27.6 [24.8;30.8]	27.6 [24.8;30.8]	26.8 [24.0;30.1]	0.97 [0.96;0.97]	<0.001
Gender:					
F	157,673 (27.2%)	154,169 (27.1%)	3504 (32.6%)	Ref.	Ref.
M	422,204 (72.8%)	414,961 (72.9%)	7243 (67.4%)	0.77 [0.74;0.80]	<0.001
Treated or BP > 140/90 mmHg on >1 occasion before admission	372,353 (64.9%)	365,084 (64.9%)	7269 (68.6%)	1.19 [1.14;1.24]	<0.001
Diabetes Mellitus:					
Not Diabetic	461,350 (79.5%)	452,928 (79.6%)	8422 (78.3%)	Ref.	Ref.
Diet	21,721 (3.74%)	21,283 (3.74%)	438 (4.07%)	1.11 [1.00;1.22]	0.043
Oral therapy	65,151 (11.2%)	63,902 (11.2%)	1249 (11.6%)	1.05 [0.99;1.12]	0.105
Insulin	31,895 (5.50%)	31,247 (5.49%)	648 (6.02%)	1.12 [1.03;1.21]	0.009
Smoking Status:					
Never smoked	211,251 (37.1%)	207,284 (37.1%)	3967 (37.6%)	Ref.	Ref.
Ex smoker	298,431 (52.4%)	292,966 (52.4%)	5465 (51.8%)	0.97 [0.94;1.02]	0.224
Current smoker	59,878 (10.5%)	58,756 (10.5%)	1122 (10.6%)	1.00 [0.93;1.07]	0.951
Extracardiac Arteriopathy:					
No	515,052 (88.8%)	506,386 (88.9%)	8666 (80.6%)	Ref.	Ref.
Yes	65,065 (11.2%)	62,974 (11.1%)	2091 (19.4%)	1.94 [1.85;2.04]	<0.001
Previous Cerebral Vascular Accident:					
No	560,619 (96.6%)	550,649 (96.7%)	9970 (92.7%)	Ref.	Ref.
Yes	19,498 (3.36%)	18,711 (3.29%)	787 (7.32%)	2.32 [2.16;2.50]	<0.001
Left Ventricular Ejection Fraction:					
Good ≥ 50%	400,752 (70.5%)	393,982 (70.7%)	6770 (64.2%)	Ref.	Ref.
Fair (LVEF 30–50%)	135,394 (23.8%)	132,418 (23.8%)	2976 (28.2%)	1.31 [1.25;1.37]	<0.001
Poor (LVEF < 30%)	31,937 (5.62%)	31,144 (5.59%)	793 (7.52%)	1.48 [1.37;1.60]	<0.001
Pre-Operative Atrial Fibrillation:					
No Atrial Fibrillation	492,076 (89.7%)	483,829 (89.7%)	8247 (84.5%)	Ref.	Ref.
Atrial Fibrillation	56,773 (10.3%)	55,257 (10.3%)	1516 (15.5%)	1.61 [1.52;1.70]	<0.001
CABG operation:					
No	171,947 (29.6%)	167,502 (29.4%)	4445 (41.3%)	Ref.	Ref.
Yes	408,170 (70.4%)	401,858 (70.6%)	6312 (58.7%)	0.59 [0.57;0.62]	<0.001
Cardiopulmonary bypass:					
No	55,332 (9.77%)	54,869 (9.87%)	463 (4.39%)	Ref.	Ref.
Yes	510,892 (90.2%)	500,812 (90.1%)	10,080 (95.6%)	2.38 [2.17;2.62]	<0.001
Aortic Valve Surgery:					
No	421,908 (72.7%)	415,069 (72.9%)	6839 (63.6%)	Ref.	Ref.
Yes	158,209 (27.3%)	154,291 (27.1%)	3918 (36.4%)	1.54 [1.48;1.60]	<0.001
Mitral Valve Surgery:					
No	512,843 (88.4%)	503,835 (88.5%)	9008 (83.7%)	Ref.	Ref.
Yes	67,274 (11.6%)	65,525 (11.5%)	1749 (16.3%)	1.49 [1.42;1.57]	<0.001
Tricuspid Valve Surgery:					
No	566,993 (97.7%)	556,568 (97.8%)	10,425 (96.9%)	Ref.	Ref.
Yes	13,124 (2.26%)	12,792 (2.25%)	332 (3.09%)	1.39 [1.24;1.55]	<0.001
Surgery on thoracic aorta:					
No	551,169 (95.0%)	542,108 (95.2%)	9061 (84.2%)	Ref.	Ref.
Yes	28,948 (4.99%)	27,252 (4.79%)	1696 (15.8%)	3.72 [3.53;3.93]	<0.001

**Table 2:** Adjusted Odds ratios (ORs) and 95%CI = Confidence Interval by the multivariate logistic regression model on the training set using Stroke After Cardiac Surgery (SACS) as dependent variable.

Variable	OR [95%CI]	*p*-Value
Age, years	1.03 [1.03–1.04]	<0.001
BMI, kg/m^2^	0.98 [0.97–0.98]	<0.001
Gender (Ref. F)		
M	0.92 [0.87–0.98]	0.008
Systemic Arterial Hypertension (Ref. No hypertension)		
Treated or BP > 140/90 mmHg on > 1 occasion before admission	1.10 [1.03–1.16]	0.003
Diabetes Mellitus (Ref. Non-Diabetic)		
Diet	1.15 [1.01–1.31]	0.033
Oral therapy	1.37 [1.23–1.53]	<0.001
Smoking Status (Ref. Never smoke)		
Ex-smoker	1.20 [0.94–1.06]	0.987
Current smoker	1.32 [1.08–1.32]	<0.001
ExtraCardiac Arteriopathy (Ref. No)		
Yes	1.70 [1.58–1.82]	<0.001
Previous Cerebral Vascular Accident (Ref. No)		
Yes	1.71 [1.54–1.89]	<0.001
Left Ventricular Ejection Fraction (Ref. Good (≥50%))		
Fair (LVEF 30–50%)	1.35 [1.22–1.49]	<0.001
Poor (LVEF < 30%)	1.24 [1.17–1.31]	<0.001
Pre-Operative Atrial Fibrillation (Ref. No)		
Yes	1.12 [1.03–1.21]	0.005
CABG operation (Ref. No)		
Yes	0.89 [0.83–0.96]	0.002
Cardiopulmonary bypass (Ref. No)		
Yes	1.78 [1.57–2.03]	<0.001
Aortic Valve Surgery (Ref. No)		
Yes	1.16 [1.08–1.23]	<0.001
Mitral Valve Surgery (Ref. No)		
Yes	1.43 [1.31–1.56]	<0.001
Tricuspid Valve Surgery (Ref. No)		
Yes	0.88 [0.75–1.04]	0.133
Surgery on thoracic aorta (Ref. No)		
Yes	3.30 [3.03–3.60]	<0.001
Ref: Reference category		

## Data Availability

No new data were created on patient features due to study design.
